# The effect of nerve growth factor on supporting spatial memory depends upon hippocampal cholinergic innervation

**DOI:** 10.1038/s41398-021-01280-3

**Published:** 2021-03-15

**Authors:** Wei Zheng Eu, Yu-Ju Chen, Wei-Ting Chen, Kuan-Yu Wu, Cheng-Yu Tsai, Sin-Jhong Cheng, Roderick N. Carter, Guo-Jen Huang

**Affiliations:** 1 Department and Graduate Institute of Biomedical Sciences, College of Medicine, Chang Gung University, Taoyuan, Taiwan; 2 Institute of Biomedical Sciences, Academia Sinica, Taipei, Taiwan; 3 British Heart Foundation Centre for Cardiovascular Science, The University of Edinburgh, Edinburgh, UK; 4 Healthy Aging Research Center, Chang Gung University, Taoyuan, Taiwan; 5 Neuroscience Research Center, Chang Gung Memorial Hospital, Linkou, Taiwan

**Keywords:** Hippocampus, Long-term memory

## Abstract

Nerve growth factor (*NGF*) gene therapy has been used in clinical trials of Alzheimer’s disease. Understanding the underlying mechanisms of how NGF influences memory may help develop new strategies for treatment. Both NGF and the cholinergic system play important roles in learning and memory. NGF is essential for maintaining cholinergic innervation of the hippocampus, but it is unclear whether the supportive effect of NGF on learning and memory is specifically dependent upon intact hippocampal cholinergic innervation. Here we characterize the behavior and hippocampal measurements of volume, neurogenesis, long-term potentiation, and cholinergic innervation, in brain-specific *Ngf*-deficient mice. Our results show that knockout mice exhibit increased anxiety, impaired spatial learning and memory, decreased adult hippocampal volume, neurogenesis, short-term potentiation, and cholinergic innervation. Overexpression of *Ngf* in the hippocampus of *Ngf* gene knockout mice rescued spatial memory and partially restored cholinergic innervations, but not anxiety. Selective depletion of hippocampal cholinergic innervation resulted in impaired spatial memory. However, *Ngf* overexpression in the hippocampus failed to rescue spatial memory in mice with hippocampal-selective cholinergic fiber depletion. In conclusion, we demonstrate the impact of *Ngf* deficiency in the brain and provide evidence that the effect of NGF on spatial memory is reliant on intact cholinergic innervations in the hippocampus. These results suggest that adequate cholinergic targeting may be a critical requirement for successful use of *NGF* gene therapy of Alzheimer’s disease.

## Introduction

Nerve growth factor (NGF), a neurotrophic growth factor first identified in the 1950s, promotes neurite outgrowth and stimulates nerve cell differentiation in culture experiments^1–3^. In the brain, the highest expression of *Ngf* mRNA is found in the hippocampus^4^. NGF activates two receptors: the low-affinity NGF receptor (common neurotrophic receptor, p75) and the high-affinity NGF receptor (TrkA), which are both expressed in basal forebrain cholinergic neurons^5,6^ and are known to innervate the hippocampal structure^7^. Evidence suggests that NGF is released from target cells and triggers signaling pathways retrogradely along the axon^8^. There is a strong link between NGF and the cholinergic system in the hippocampus. Injecting NGF into the lateral ventricles increases choline acetyltransferase (ChAT) activity in the hippocampus^9^. Furthermore, NGF has been shown to protect basal forebrain cholinergic neurons after axonal damage of hippocampal projecting fibers^10^.

Several hypotheses have been proposed for the cause of Alzheimer’s disease. One of them is the NGF hypothesis^11^. Since the early 1980s, several reports have shown that significant cholinergic dysfunction occurs in demented elderly people, and that cholinergic neuronal degeneration associates with cognitive deficits in Alzheimer’s disease^12^. The novel positron emission tomography (PET) radiotracer ^18^F-fluoroethoxybenzonesamicol has provided more recent evidence that there is more cortical cholinergic terminal loss in patients with Alzheimer’s disease^13^. Animal studies also provide evidence that the density of cholinergic innervations decrease in aged rats and Alzheimer’s disease-like transgenic mice^14,15^. Moreover, aged anti-NGF mice exhibit Alzheimer’s disease-like phenotypes, such as neuronal loss, cholinergic deficits, and impairments in spatial memory^16^. A recent study suggests that the NGF metabolic pathway is impaired in patients with Alzheimer’s disease, finding that the molecular machinery for proNGF maturation is reduced while that for mature NGF degradation is enhanced^17^.

The central cholinergic system is involved in learning^18,19^ and NGF appears to play an important role in supporting this. Infusion of NGF intraventricularly can rescue both mild cholinergic depletion in the hippocampus and memory deficits in heterozygous *Ngf*^*+/−*^ mutant mice^20^. Evidence also shows that NGF modulates the strength of cholinergic projections to the hippocampus and affects neuronal plasticity and retention of spatial learning behavior^21^. However, it remains unknown whether the effects of NGF on learning depend on these cholinergic projections to the hippocampus. Clarification of this may provide a better understanding for developing treatment for Alzheimer’s disease. In this study, we addressed this question by using brain-specific *Ngf* conditional knockout (cKO) mice to reveal the importance of NGF in anxiety and spatial memory. Furthermore, we found that the effect of NGF on spatial memory, but not anxiety, requires the cholinergic projection to the hippocampus.

## Materials and methods

### Animals

Experiments were performed on sex-balanced cohorts of 12-week-old *Ngf* cKO and wild-type (WT) littermate mice. *Ngf* floxed mice from the National Laboratory Animal Center, Taiwan, (RMRC 13175) and *Sox1::Cre* mice (Acc. No. CDB0525K)^22^ were used to generate *Ngf* ^flox/flox^*;Sox1::Cre* mice in this study. *Ngf* ^flox/flox^;*Sox1::Cre* (cKO) and *Ngf* ^flox/flox^ (WT) mice used in this study were littermates generated by crossing *Ngf* ^flox/flox^;*Sox1::Cre* mice with homozygous *Ngf* ^flox/flox^ mice. Twelve-week-old male C57BL/6Narl mice were used for hippocampal cholinergic denervation. Mice were bred in the AAALAC-certified specific pathogen-free conditions. They were housed in a 12 : 12 h light–dark cycle at a temperature of 22 °C and a humidity level of 60–70%. Animals had ad libitum access to food and water. All procedures were carried out in accordance with the local regulations and were approved by the Institutional Animal Care and Use Committee at Chang Gung University (Permit Number: CGU105-033).

### Behavioral tests

For behavioral tests, the movement of animals was recorded and tracked using a video recording system. Mice for conducting the behavioral analysis were randomized to a separate cage for testing, with bedding, food, and water, before being transferred back to the home cage. All mazes are made of acrylic; there is no bedding in the maze during the test. All the behavioral data were recorded and acquired automatically by Ethovision software.

#### Open field

Mice were put into a round open field with radius = 45 cm and inner circle with radius = 30 cm. They were allowed to move freely for 5 min and were tracked by Ethovision software.

#### Elevated plus maze

The apparatus used for the elevated plus maze (EPM) test consisted of two open arms (30 × 5 cm) and two enclosed arms of the same size, with 15 cm-high walls. The maze was elevated 38 cm above the floor. Mice were placed in the EPM for 5 min with dim light and the time spent in the open arm was measured.

#### Morris water maze

This was conducted in a round water maze tank (110 cm in diameter, 50 cm deep) located in a bright room. The pool was filled to a depth of 15 cm with water made opaque by adding white, non-toxic paint. A square escape platform (9 × 9 cm) was submerged 0.5 cm below the water surface, in a fixed position in one of the quadrants. Mice were trained over 4 days. On each training day, mice received four training trials. The inter-trial interval was 15 min. On each trial, mice were placed into the pool at one of four starting locations. Mice were allowed to swim/search for a maximum of 90 s or until they found the platform, and were allowed to remain on the platform for 30 s. If the mouse failed to find the platform on a given trial, the mouse was guided to the platform. Eight days after the last trial, for the memory test, the platform was removed from the pool and the mouse was allowed 90 s to search for it. Data acquisition and analysis were performed automatically, including the swimming path length of training trials and the percentage time spent in the target quadrant for the memory test.

#### Novel object recognition

Mice were allowed to familiarize with two identical white acrylic cylinders (height = 9 cm, diameter = 5 cm) for 15 min per day, for 2 days. The objects were in a fixed location in a white chamber. During the test phase on the third day, one of the exposed objects was replaced with a gray shot glass (height = 8.7 cm, diameter = 5 cm) fixed upside down in the chamber and the other object was replaced with a new but identical white acrylic cylinder. Time spent exploring each of the objects was collected and analyzed by Ethovision software. A discrimination index (DI) for each test was calculated by: DI = (duration_novel object_ − duration_familiar object_)/(duration_novel object_ + duration_familiar object_).

### Hippocampal volumetric analysis

All magnetic resonance imaging (MRI) scans were performed using ClinScan 70/30 USR (Bruker BioSpin, Ettlingen, Germany) with a phased array coil tailored for the mouse head. A three-dimensional (3D) gradient echo sequence was chosen to acquire brain multi-slice images with the following parameters: repetition time = 30 ms; echo time = 15 ms; flip angle = 20°; number of averages = 5; slice thickness = 0.2 mm; in-plane resolution = 256 × 256; voxel size = 59 × 59 × 200 μm^3^. The total acquisition time was around 15 min with 1 ~ 2% Isoflurane as anesthetic. Hippocampal volume was measured with PMOD 3.2 (PMOD Technologies, Switzerland). Hippocampi were manually outlined across 13 or 14 coronal slices per animal; the volume of each slice was obtained by multiplying the outlined area to the slice thickness (0.2 mm). The total brain volume was measured using manually outlined areas across 19 sagittal slices per animal, multiplying the outlined area to the slice thickness (0.2 mm). Images were analyzed under the same conditions in a blinded manner.

### mRNA quantification

Hippocampal tissue was collected from WT and cKO mice, then homogenized in Trizol, followed by phenol–chloroform extraction of total RNA. cDNA was then made using SuperScript III reverse transcriptase (Invitrogen). Experiments were performed in duplicate. Gene expression levels were then calculated with the ΔΔCt method and normalized against a *Gapdh* control. *Ngf*-Forward: 5′-TTCTA TACTG GCCGC AGTGA-3′; *Ngf*-Reverse: 5′-TTAGT CCAGT GGGCT TCAGG-3′.

### Corticosterone assay

Blood samples were collected between 7:30 and 8:00 p.m. by facial vein puncture and were collected into Microvette^®^ CB 300 LH tubes. Plasma was separated from whole blood by centrifugation and was stored in centrifuge tubes at −80 °C until analyzed. To analyze, samples were diluted in buffer (1/40), then analyzed by a Corticosterone EIA kit (Enzo Life Sciences) according to the manufacturer’s instructions, as previously described^23^.

### Viral vector preparation and intra-hippocampal injections

For the *Ngf* overexpression experiment, DNA fragments that encoded *Ngf* (RefSeq NM_013609) or enhanced Green Fluorescent Protein (eGFP) were created by PCR and subcloned into the NotI site of an AAV9 virus construct. Recombinant AAV9 vectors were produced by a standard triple-plasmid transfection method and were purified by two rounds of CsCl centrifugation. The physical vector titers of AAVs were quantified by measuring the number of packaged vector genomes using a real-time PCR method.

For the viral vector injections, surgery was performed under anesthesia (a Zoletil-Rompun mixture). Mice received bilateral injections of viral vectors into the hippocampus in a volume of 1.5 µl under stereotaxic guidance. There were four injection sites in total (for dorsal hippocampus, Anterior-Posterior (AP) −2.2 mm, Medial-Lateral (ML) ±2.0 mm from the bregma, and Dorsal-Ventral (DV) −1.8 mm from the dura; for ventral hippocampus, AP −3.0 mm, ML ±3.0, and DV −3.3 mm). The final concentration of the injected AAV vector was 3.1 × 10^12^ vg/ml.

### Immunohistochemistry/in situ hybridization and quantification

All sections for labeling of Ki67, Doublecortin (DCX), 5-bromo-2’-deoxyuridine (BrdU)/NeuN, and ChAT were sliced to a thickness of 40 µm on a sliding microtome. Sections were mounted on SuperFrost slides and dried overnight. Subsequently, slides were incubated in 0.01 mol/L citric buffer for 20 min at 90 °C, 3% H_2_O_2_ for 10 min, rinsed in phosphate-buffered saline (PBS), and incubated overnight at room temperature with rabbit anti-Ki67 antibody (1 : 3000, Abcam), rabbit anti-DCX antibody (1 : 4000, Abcam), and anti-ChAT antibody (1 : 200, Abcam). The next day, a standard rabbit IgG ABC kit (Vector Lab) procedure was used and the slides reacted for 5–10 min with a Sigma DAB tablet. Sections were then cover-slipped with DPX.

For BrdU/NeuN double labeling, sections were incubated in 2 N HCl for 15 min at 37 °C, neutralized in Sodium Borate (Sigma) for 10 min (pH 8.5), and washed three times in PBS before incubation with rat anti-BrdU (1 ∶ 1500, Abcam) and mouse anti-NeuN (1 ∶ 1000, Millipore). Following three washes in PBS (5 min each), sections were incubated with the fluorescent secondary antibody (1 ∶ 250, Alex Fluor 488 and Texas Red, Invitrogen) for 2 h in 0.3% Triton/PBS with 2% of goat serum.

For *Ngf* in situ hybridization, *Ngf* probe (Catalog number 565131) and RNAscope 2.5 HD Brown Reagent Kit (Advanced Cell Diagnostics, USA) were used, and staining was performed according to the product’s manual. Briefly, slides were incubated in 1× retrieval buffer for 5 min at 100 °C, 3% H_2_O_2_ for 10 min, and rinsed in 1× wash buffer. Slides were then incubated in protease for 20 min and, subsequently, target probe for 2 h at 40 °C. After a series of amplification, DAB was used to visualize the RNA.

DCX, Ki67-labeled, and BrdU/NeuN double-labeled cells were counted on every eighth section through the entire rostrocaudal extent of the granule cell layer (six sections per animal). The number of cells counted was then multiplied by 16, to obtain an estimate of the total number of DCX, Ki67, and BrdU-positive cells in the dentate gyrus. All slides were randomized and coded before quantitative analysis. ChAT-labeled cell somata were counted on every second section through the rostrocaudal extent of the nucleus of the vertical limb of the diagonal band (VDB) and medial septum (MS) (four sections per animal). For representative image of BrdU/NeuN double labeling, *z*-stack image (seven slices in stack) was first acquired under ×40 objective, followed by 3D deconvolution with fast iterative mode using Axiovision Microimaging software (Carl Zeiss).

The cholinergic fibers were visualized in ChAT staining and examined in dark field, under ×10 objective (Axio Imager 2, captured by Nikon D5100). The fiber density was quantified as mean intensity (Mean Gray Value) of thresholded signal under the hippocampal area with ImageJ 1.42q (NIH). Hippocampal cholinergic fiber density was quantified unilaterally on every eighth section through the entire rostrocaudal extent of the whole hippocampus.

### Electrophysiology in hippocampal slices

The hippocampal slices (450 µm thickness) were transverse cut with a vibrating tissue slicer (D.S.K. Microslicer DTK-1000; Dosaka EM, Kyoto, Japan). The slices were allowed to recover for at least 120 min and were transferred to an immersion-type recording chamber mounted on an upright microscope (BX51WI; Olympus Optical, Kyoto, Japan) equipped with an infrared-differential interference-contrast microscopic video. Oxygenated artificial cerebrospinal fluid (5% CO_2_/95% O_2_) containing 0.1 mM picrotoxin was perfused into the recording chamber at a rate of 1–2 ml/min. A bipolar stainless steel stimulating electrode (Frederick Haer Company, Bowdoinham, ME) (10 Meg-ohm impedance) and a glass pipette filled with 3 M NaCl were positioned in the stratum radiatum of CA1 for evoking and recording the field excitatory postsynaptic potentials (fEPSPs) activity, respectively. Paired-pulse facilitation (PPF) was investigated at various interstimulus intervals and each interstimulus interval applied two short-duration current pulse (40 μs) every 20 s for at least four times. The ratios of initial slopes from second fEPSP response/first fEPSP response were measured for data analysis. For long-term potentiation (LTP) experiments, stable baseline fEPSP activity was evoked every 20 s for at least 20 min, high-frequency stimulation (HFS)-LTP was then induced using 5 trains of 100 pulses at 100 Hz with inter-train interval of 20 s. Electrophysiological traces were amplified with an amplifier (Multiclamp 700 B; Axon Instruments, Union City, CA). All signals were low-pass filtered at 1 kHz and digitized at 10 kHz using a CED Micro 1401 mKII interface (Cambridge Electronic Design). Data were collected using Signal software (Cambridge Electronic Design, Cambridge, UK). Synaptic responses were normalized to the average of the baseline. Short-term potentiation (STP) and LTP values were determined by averaging 5 min of normalized slope values at 10–15 min and 55–60 min post HFS, respectively. Experimenters who conducted the electrophysiology were blinded to the mice group.

### Hippocampal cholinergic denervation

Murine p75-antibody with Saporin conjugated (mu p75-SAP) (ATS Bio, San Diego) was used in cholinergic denervation. Then, 0.2 μg of the mu p75-SAP was administered per site and the injection sites were similar to the viral vector injection sites (four injection sites per animal).

### Statistical analysis

The mean ± SEM was determined for each group. Statistical analysis was performed using Graphpad Prism software. Data were analyzed via an analysis of variance or t-test as appropriate. Bonferroni method was performed when applicable. Differences were considered significant when *p* was <0.05.

## Results

### Decreased hippocampal neurogenesis, cholinergic fiber density, and volume in nervous system-specific *Ngf* KO mice

**Fig. 1:**
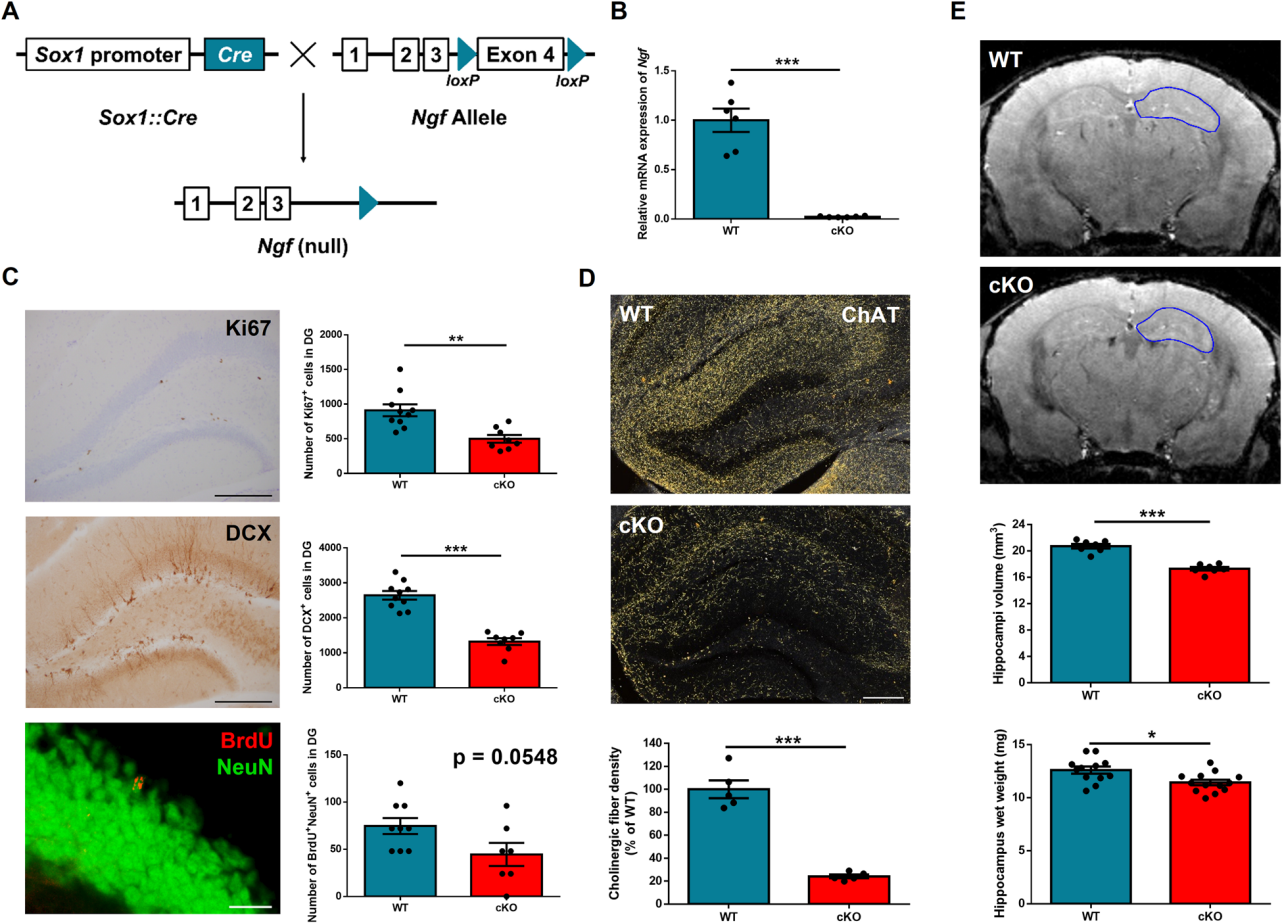
*Ngf*-knockout mice show decreased hippocampal neurogenesis and depleted cholinergic innervations. **A** *Ngf* floxed mice were crossed with *Sox1*::*Cre* mice to knock out *Ngf* specifically in the brain from early development. **B** We confirmed the high knockout efficiency of *Ngf* in the hippocampus at mRNA (*n* = 6). **C** Images of neurogenesis markers DCX, Ki67, and BrdU and cell counts in the dentate gyrus of the hippocampus. There were significantly decreased cell counts of Ki67- and DCX-positive cells in the *Ngf* cKO hippocampus (*n* = 8–10). For the BrdU/NeuN-positive cell counts, *Ngf* cKO mice also exhibited decreased cell counts (*n* = 7–9). **D** Images and quantification of ChAT in the hippocampus. *Ngf* cKO mice showed depleted cholinergic fiber in the hippocampus (*n* = 5). **E** Representative images of MRI scans and quantification of hippocampal volume (*n* = 7). *Ngf* cKO hippocampi are smaller in volume compared to WT. Decreased hippocampal weight in *Ngf* cKO mice were consistent with the result from the MRI (*n* = 12–13). Data shown as mean wet weight of bilateral hippocampus.

A major challenge to the study of the functions of NGF in adult mice is that conventional KO models die prematurely^24^. So far, most reports have used NGF blockers, such as small interfering RNA or antibodies, to evaluate the effect of NGF^21,25,26^. To study the role of NGF in the brain, we crossed *Ngf* floxed mice with *Sox1*::*Cre* mice, which express *Cre* throughout the neural tube from E11^27^. These tissue-specific cKO mice allow us to investigate the functions of *Ngf* in the brain (Fig. 1A). First, hippocampal *Ngf* mRNA expression was measured by quantitative PCR. The results show a robust decrease of *Ngf* expression in cKO mice (*p* < 0.0001, *t*_(10)_ = 8.24) (Fig. 1B). To further confirm the specificity of this brain cKO, we measured *Ngf* mRNA levels in the whole brain, liver, and muscle from WT and cKO mice, and our results confirm the specificity of KO (Supplementary Fig. S1a).

NGF infusion into lateral ventricles increases both the survival of newly born neurons and cholinergic activity in the hippocampus^28^. We therefore asked whether *Ngf*-deficient mice have lower levels of adult hippocampal neurogenesis and cholinergic innervation. To detect adult neurogenesis, we injected BrdU (200 mg/kg, one injection, intraperitoneally) into WT and *Ngf* cKO adult mice (around 13 weeks old). Mice were killed 34 days after this BrdU injection to evaluate neuronal survival. We stained brain tissue for Ki67 (a cell proliferation marker), DCX (marker for immature neurons), and double staining for BrdU and NeuN (neuronal marker). To assess cholinergic fiber density, we stained for ChAT, the enzyme responsible for the synthesis of acetylcholine. We detected significantly less Ki67 (*p* = 0.0016, *t*_(16)_ = 3.8) and DCX (*p* < 0.0001, *t*_(16)_ = 8.14) positive cell number in the dentate gyrus of the hippocampus in *Ngf* cKO mice compared to WT mice. For BrdU and NeuN double labeling, *Ngf* cKO mice also showed a trend for lower level of BrdU+/NeuN+ cell number (*p* = 0.054, *t*_(14)_ = 2.09) (Fig. 1C). Together, these data demonstrate that a deficit of NGF in the brain results in decreased adult hippocampal neurogenesis. Plasma corticosterone is a major factor in the control of adult neurogenesis^29^, and so to determine whether the decreased neurogenesis in *Ngf* cKO mice is due to elevated plasma corticosterone, we measured the basal level of plasma corticosterone from WT and cKO mice. There was no significant difference in basal plasma corticosterone (*p* = 0.24, *t*_(9)_ = 1.25), suggesting that the effect of *Ngf* on neurogenesis is not caused by differential in plasma corticosterone (Supplementary Fig. S1b).

Previous work has shown that cholinergic projections to the hippocampus are from the basal forebrain^30^. Thus, we assessed whether there are any changes to cholinergic septohippocampal projections in *Ngf* cKO mice. Our results from ChAT staining show that the density of cholinergic fibers is dramatically decreased in the hippocampus of *Ngf* cKO mice (around 76% decreased, *p* < 0.0001, *t*_(8)_ = 9.56) (Fig. 1D), but not other cortical regions, such as medial prefrontal cortex (*p* = 0.12, *t*_(8)_ = 1.73) and temporal association cortex (*p* = 0.2, *t*_(16)_ = 1.31) (Supplementary Fig. S2a, b). Together, these data indicate that NGF is required for supporting the cholinergic projections to the hippocampus, but not the cortex. Next, we analyzed the cholinergic neuronal cell count in the VDB of Broca and MS, from where it projects cholinergic fibers to the hippocampus^31,32^. *Ngf* cKO mice display a small decrease (around 20% decreased) in the ChAT cell counts in MS and VDB (*p* = 0.022, *t*_(16)_ = 2.52) (Supplementary Fig. S2c).

During sectioning of the brain tissue, we found that the hippocampus of cKO mice appeared to be small. To confirm this, we conducted an MRI scan and our results confirm that *Ngf* cKO have smaller hippocampi than WT (*p* < 0.0001, *t*_(12)_ = 12). To further verify this, we dissected the hippocampus and measured the weight, and we found the hippocampi of cKO mice were lighter in weight compared to WT mice (*p* = 0.01, *t*_(23)_ = 2.79) (Fig. 1E).

### Decreased anxiety response and spatial learning in *Ngf* cKO mice

Blockade of endogenous NGF impairs retention of spatial memory^21^ and disrupts cholinergic signaling, whereas deletion of the forebrain cholinergic system influences anxiety and spatial learning^18,33^. To better understand the effects of *Ngf* cKO in anxiety and learning, we subjected mice to open field, EPM, water maze testing, and novel object recognition.

**Fig. 2:**
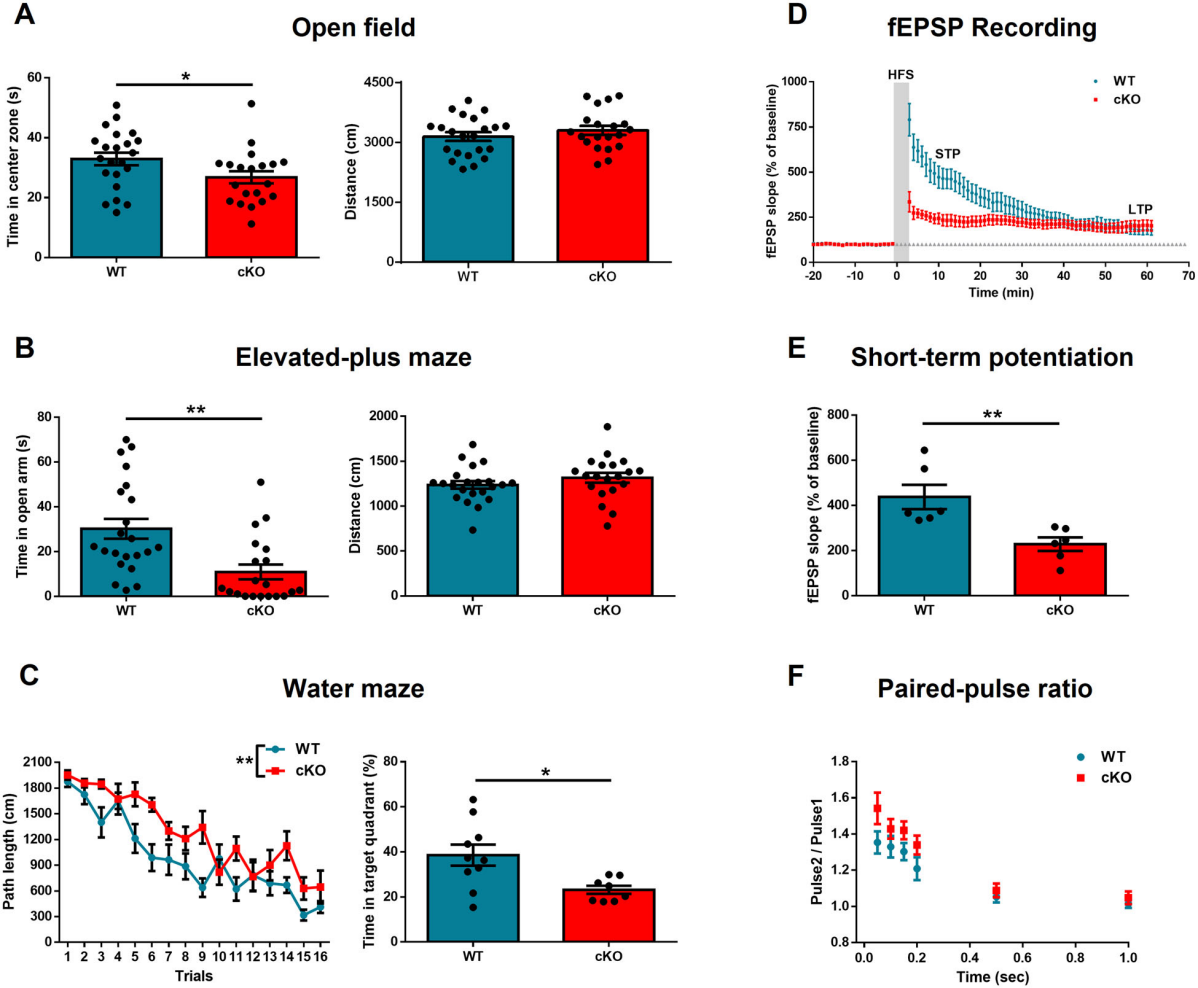
*Ngf*-knockout mice show increased anxiety and impaired spatial learning. **A** *Ngf* cKO mice spent less time in the center zone of the open field, but there was no difference in the distance traveled (*n* = 20–22). **B** In the elevated plus maze, *Ngf* cKO mice spent less time in the open arm but there was no difference in the distance traveled. **C** In the water maze, *Ngf* cKO mice exhibited impaired learning in the training section. One week later, *Ngf* cKO mice spent less time in the target zone (*n* = 8–10). **D** High-frequency stimulation-induced synaptic plasticity was recorded in the hippocampal CA1 region in WT and *Ngf* cKO mice (*n* = 6). The horizontal gray line indicates the average value of the normalized amplitude during the baseline period. **E** fEPSP slope of STP was dramatically reduced in the *Ngf* cKO group. **F** The paired-pulse ratios were similar between *Ngf* cKO and WT mice; however, *Ngf* cKO exhibited a trend of enhanced PPR at the use of a 50 ms interstimulus interval.

*Ngf* deletion increased anxiety-like behavior and decreased spatial learning and memory, without affecting the novel object recognition. In the open-field test, cKO mice spent less in the center zone (*p* = 0.04, *t*_(40)_ = 2.09), with no difference in total distance traveled (*p* = 0.31, *t*_(40)_ = 1.01) (Fig. 2A). In the EPM, cKO mice spent more time in the open arm (*p* = 0.001, *t*_(40)_ = 3.43) but they did not exhibit a significant difference in total distance (*p* = 0.25, *t*_(40)_ = 1.15) (Fig. 2B). In the water maze test, *Ngf* cKO mice performed worse in training section (*p* = 0.001, *F*_(1,16)_ = 15.65). One week later, we performed a memory test. Mice were placed in the water maze but with the platform removed. *Ngf* cKO mice spent less time in the target quadrant (*p* = 0.01, *t*_(16)_ = 2.79) (Fig. 2C). For novel object recognition, there is no difference in the DI (*p* = 0.41, *t*_(20)_ = 0.82). Together, these data indicate that NGF plays a role in mediating anxiety and spatial learning/memory.

Hippocampal LTP has been widely considered a synaptic model of memory^34^ and direct intra-septal infusions of NGF facilitate the induction of LTP^21^. To examine the effect of NGF on synaptic plasticity, we measured LTP through ex vivo electrophysiological recording in hippocampal CA1 Schaffer collaterals pathway of brain slices in WT and *Ngf* cKO mice. Our results revealed that HFS produced a persistent increase in the slope of fEPSPs in CA1 neurons of both WT and *Ngf* cKO mice, but no difference in LTP (Fig. 2D). However, after five trains of HFS tetanizing stimulus, the STP were significantly decreased in the *Ngf* cKO group (10–15 min after HFS, *p* = 0.006, *t*_(10)_ = 3.39) (Fig. 2E). To further confirm the effect on presynaptic transmission, we examined the short-term synaptic plasticity by PPF. The paired-pulse ratios (PPRs) were similar between *Ngf* cKO and WT mice. However, we observe a trend of enhanced PPR from *Ngf* cKO samples using a 50 ms interstimulus interval (*p* = 0.094, *t*_(18)_ = 1.76) (Fig. 2F), suggesting that the presynaptic transmission may have been changed in the *Ngf* cKO mice.

### Overexpression of *Ngf* in the hippocampus of cKO mice rescues spatial memory, but not anxiety

**Fig. 3:**
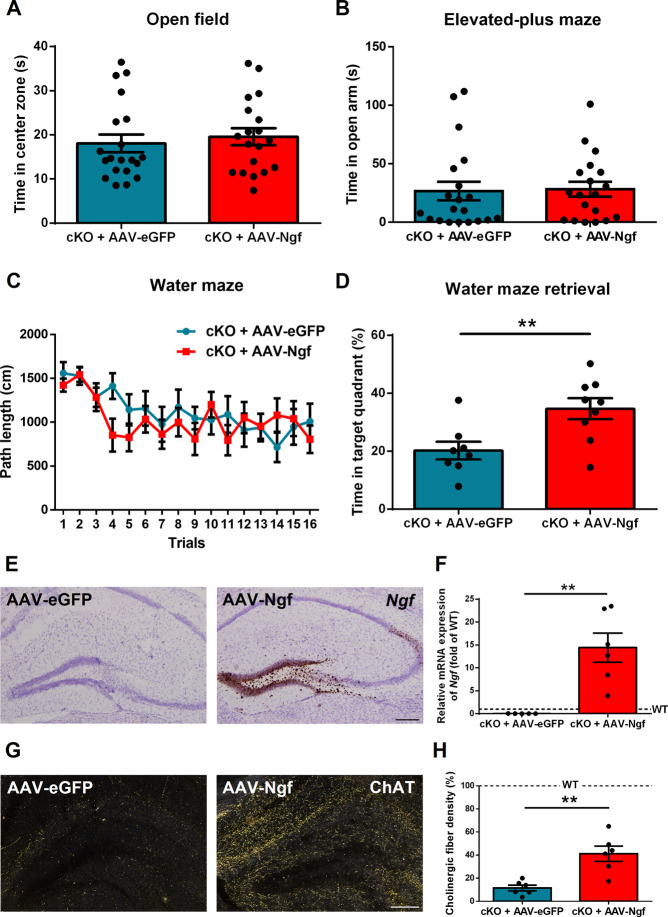
Overexpression of hippocampal *Ngf* in knockout mice restore cholinergic innervations and spatial memory. **A**, **B** Hippocampal *Ngf* overexpression (using AAV-*Ngf*) on the *Ngf* cKO background had no effect on the open field or elevated plus maze (*n* = 19–20). **C**, **D** *Ngf* overexpression did not improve spatial learning (*n* = 8–9), but did improve spatial memory retention. **E** Images of *Ngf* overexpression in the hippocampus by in situ hybridization. **F** We confirm the *Ngf* overexpression in the hippocampus by real-time PCR (*n* = 5–6). **G**, **H** Images of cholinergic fibers in AAV-eGFP control and AAV-*Ngf* mice by ChAT staining. Quantification of fiber density revealed that *Ngf* overexpression partially restored cholinergic fibers in the hippocampus (*n* = 6). Dotted lines represent the levels of age-matched WT naive mice.

To determine whether the behavioral phenotypes we observed in *Ngf* cKO mice can be reversed by overexpressing *Ngf* in the hippocampus, we injected AAV-CB (chicken β-actin)-*Ngf* viral vector (overexpression) or AAV-CB-eGFP viral vector (control) into the hippocampus of *Ngf* cKO mice. Fourteen days after surgery, we subjected all mice to behavioral testing. Results show no significant difference in open-field test (time in center zone, *p* = 0.59, *t*_(37)_ = 0.54) and EPM (time in open arm, *p* = 0.76, *t*_(37)_ = 0.29) (Fig. 3A, B). For the water maze, no difference was found in the training section (*p* = 0.54, *F*_(1,15)_ = 0.39). However, cKO mice with *Ngf* overexpression spent more time in the target zone compared to the control group (*p* = 0.008, *t*_(15)_ = 3.01) (Fig. 3C, D). After harvesting the brain tissue, we confirmed *Ngf* overexpression by in situ hybridization and real-time PCR (Fig. 3E, F). We also detected a significantly higher level of cholinergic fibers in the hippocampus of *Ngf*-overexpressing mice (*p* = 0.001, *t*_(10)_ = 4.19) (Fig. 3G, H). These results suggest that *Ngf* cKO mice with hippocampal *Ngf* overexpression can restore cholinergic innervations and spatial memory, but not anxiety.

### Impaired spatial memory in mice with cholinergic denervation in the hippocampus

The *Ngf* cKO mice displayed severely decreased hippocampal cholinergic fiber innervation, which was restored by hippocampal *Ngf* overexpression. This correlated with a restoration of spatial memory performance (without an improvement of anxiety). We therefore addressed whether depletion of cholinergic fibers selectively in the hippocampus influences anxiety or spatial memory in the water maze.

**Fig. 4:**
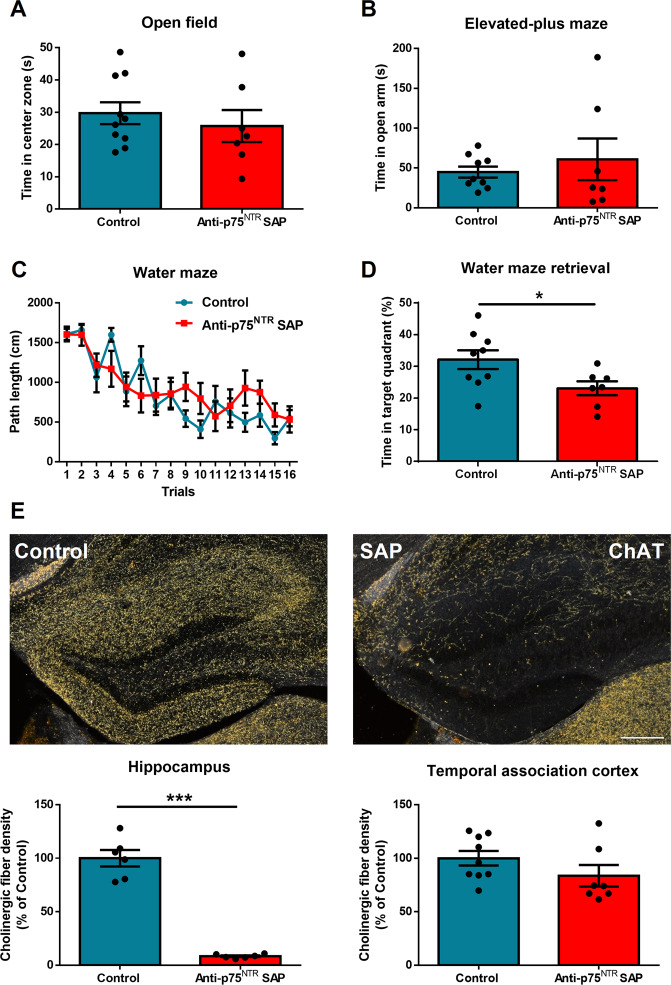
Hippocampal cholinergic depletion causes impaired spatial memory but no effect on anxiety. **A**, **B** C57 Mice injected in the hippocampus with anti-p75^NTR^Saporin to deplete cholinergic fibers (hippocampal cholinergic depletion) were not different to controls on the open field or elevated plus maze (*n* = 7–10). **C**, **D** Hippocampal cholinergic depletion did not effect spatial learning but impaired spatial memory. **E** Images of ChAT staining in PBS or anti-p75^NTR^Saporin-injected hippocampus. We confirmed that mice with anti-p75^NTR^Saporin injection had a profound decrease in cholinergic fiber density in the hippocampus without affecting the cortex (*n* = 6–9).

A previous report has shown that anti-p75^NTR^Saporin is a specific cholinergic immunotoxin in mice^35^. To this end, we injected anti-p75^NTR^Saporin (or PBS vehicle as control) into the hippocampus of C57 mice. Fourteen days after surgery, we subjected these two groups of mice to behavioral testing. We found no significant difference in both open-field test (time in center zone, *p* = 0.5, *t*_(15)_ = 0.68) and EPM (time in open arms, *p* = 0.51, *t*_(14)_ = 0.66) (Fig. 4A, B). For the water maze, no difference was found in the training section (*p* = 0.49, *F*_(1,14)_ = 0.49); however, mice injected with anti-p75^NTR^Saporin in the hippocampus spent less time in the target zone compared to the PBS control (*p* = 0.035, *t*_(14)_ = 2.33) (Fig. 4C, D). After harvesting the brain tissue, we confirmed cholinergic denervation had occurred in the hippocampus by ChAT staining (*p* < 0.0001, *t*_(10)_ = 11.85), also showing that the cholinergic fibers in the cortex were spared (*p* = 0.184, *t*_(14)_ = 1.39) (Fig. 4E). These results demonstrate that the hippocampal cholinergic system is required for maintaining spatial memory function, without having an impact on anxiety.

### Effect of NGF on spatial memory depends on hippocampal cholinergic system

**Fig. 5:**
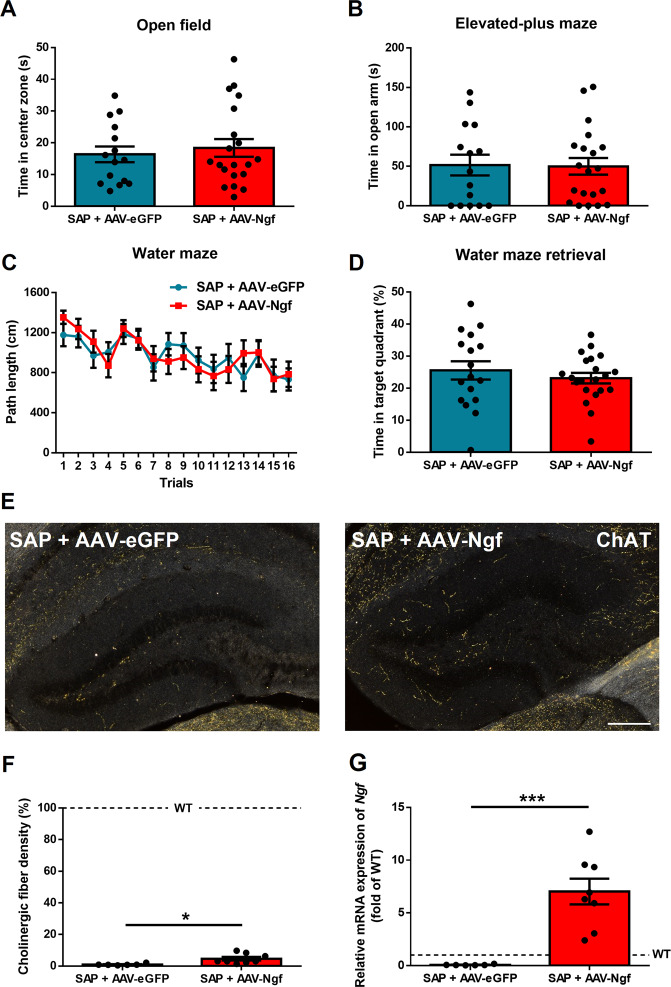
Overexpression of hippocampal *Ngf* in knockout mice with cholinergic depletion cannot rescue spatial memory. **A**, **B** Hippocampal *Ngf* overexpression (AAV-*Ngf*) in WT mice that had hippocampal cholinergic depletion using anti-p75^NTR^Saporin had no effect on the open field or elevated plus maze compared to hippocampal cholinergic-depleted mice given AAV-eGFP injection as control (*n* = 15–20). **C**, **D** *Ngf* overexpression in hippocampal cholinergic-depleted mice did not affect spatial leaning or memory (*n* = 19–21). **E**, **F** ChAT staining confirmed cholinergic depletion in both groups. **G** We confirmed the *Ngf* overexpression by real-time PCR (*n* = 6–8). Dotted lines represent the levels of age-matched WT naive mice.

We next addressed whether the spatial memory benefit gained by overexpressing *Ngf* in the hippocampus of *Ngf* cKO mice is dependent upon intact hippocampal cholinergic innervation. We injected both anti-p75^NTR^Saporin and AAV-*Ngf* viral vector into the hippocampus of *Ngf* cKO mice. For the control group, we injected both anti-p75^NTR^Saporin and AAV-eGFP viral vector. Fourteen days after surgery, we assessed both anxiety and spatial memory. Results showed no difference between AAV-*Ngf* and AAV-eGFP in the open-field test (time in center zone, *p* = 0.6, *t*_(33)_ = 0.51) and EPM (time in open arms, *p* = 0.92, *t*_(33)_ = 0.09) (Fig. 5A, B). For spatial learning and memory, there were also no differences between groups, in both the training section (*p* = 0.94, *F*_(1,36)_ = 0.004) and memory test (*p* = 0.44, *t*_(36)_ = 0.77) (Fig. 5C, D). After harvesting the brain tissue, we assessed the hippocampal cholinergic fiber density by ChAT staining and both groups showed severe depletion of hippocampal cholinergic fibers, although a slightly higher level of fiber density was found in AAV-*Ngf* mice compared to AAV-eGFP mice (*p* = 0.01, *t*_(12)_ = 2.83) (Fig. 5E, F). Real-time PCR confirmed that the AAV-*Ngf* group had dramatically increased *Ngf* expression compared to the control group (*p* = 0.0004, *t*_(12)_ = 4.89) (Fig. 5G). These data suggest that the beneficial effect of NGF on spatial memory requires intact hippocampal cholinergic innervation.

## Discussion

In this study, we set out to investigate the roles of hippocampal NGF and the cholinergic system on anxiety, learning, and memory. We showed that mice with brain-specific *Ngf* deletion (*Ngf* cKO) present with increased anxiety and a deficit in spatial learning. The *Ngf* cKO mice also presented with lower levels of adult neurogenesis, smaller hippocampal volume, impaired STP, and a dramatic depletion of cholinergic innervations in the hippocampus. Overexpression of *Ngf* in the hippocampus of *Ngf* gene KO mice rescued spatial memory but did not increase anxiety. Interestingly, we also observed a partial restoration of cholinergic innervation in the hippocampus following *Ngf* overexpression in this structure. To address whether the effect of NGF on spatial memory depends upon these cholinergic innervations, we first depleted hippocampal cholinergic fibers in WT C57 mice by injecting a cholinergic immunotoxin into the hippocampus and confirmed that hippocampal cholinergic fibers support spatial memory. Next, on *Ngf* cKO background mice, we depleted hippocampal cholinergic fibers but increased *Ngf* expression simultaneously by using AAV-*Ngf*, which were compared to similar cholinergic-depleted mice but injected with the AAV-eGFP control. The result showed that re-expression of high levels of *Ngf* in the hippocampus did not improve spatial memory. Thus, we show that the restorative effect of NGF in the *Ngf* cKO is dependent upon intact hippocampal cholinergic innervations and not by another mechanism.

Exogenously provided NGF has been shown to increase the survival of newly born neurons^28^ and spatial learning^36^ in rodents, whereas NGF blockade by antibodies impairs spatial memory^21^. Consistent with a supportive role for NGF in these parameters, we showed that the central nervous system-specific deletion of *Ngf* display a reduction of cell proliferation and neurogenesis. In behavior tests, we also observed an impairment of anxiety and spatial learning/memory.

A previous *Ngf* KO study provided evidence showing that NGF is dispensable for learning and memory^37^. However, the authors did not conduct a probe test (memory section) and instead reverse learning was performed. Furthermore, 60% of hippocampal cholinergic fibers still remained in the *Ngf*^f/f^*–Nes::Cre* mice in that study. In our mice model, we detect only 20% of cholinergic fibers left in the *Ngf*^f/f^*–Sox1::Cre* mice. This discrepancy may explain the difference in behavior we observed, as our experiment provides evidence that hippocampal cholinergic innervations are required for the effect of NGF on spatial memory. The anxiety phenotype we observed in *Ngf* cKO mice cannot be reversed by overexpressing hippocampal *Ngf*. One possibility for this is that the anxiety phenotype we observed in *Ngf* cKO mice was a consequence of *Ngf* inactivation at an early stage. Alternatively NGF may be acting outside the hippocampus to modulate anxiety. However, we do observe the regeneration of cholinergic fibers and an improvement of the retention of spatial memory in hippocampal *Ngf*-overexpressing mice, and this result is consistent with an earlier study in aged rats^38^.

We found that the cholinergic lesion of the hippocampus did not affect anxiety but impaired spatial memory. Our results are inconsistent with previous reports that showed microinfusion of an acetylcholinesterase inhibitor or muscarinic M1 and M2 receptor antagonists into the hippocampus modulate anxiety^39,40^. However, our results are consistent with a report that found no effect on anxiety following a reduction in hippocampal cholinergic innervation that was achieved by injection of immunotoxin in the MS and the VDB of Broca^41^. For the spatial learning and memory, several studies suggest that ChAT activity associates with spatial learning^18,35,42,43^, but other works showed no effect in spatial learning and memory after immunotoxin lesion^44,45^. In our experiment, we found that depleting hippocampal cholinergic innervation impaired spatial memory, without effecting acquisition. These discrepancies between studies could be due to the different behavioral protocols, age, strain, and methods to inhibit the cholinergic system in hippocampus.

An interaction between NGF and the cholinergic system has long been studied. Loss of *TrkA*, as well as the deletion of *Ngf*, reduces cholinergic neurons in the MS and cholinergic innervations in the cortical regions and hippocampus^37,46^. Similar results were observed in our *Ngf*-deficient model with around 76% of the hippocampal cholinergic fibers being denervated. To know whether these cholinergic fibers contributed to the effect of NGF in spatial learning, we increased *Ngf* expression, meanwhile depleting cholinergic innervations in *Ngf* cKO mice via intra-hippocampal injection with mixed AAV-*Ngf* (or AAV-eGFP for control group) and anti-p75^NTR^Saporin. The results show that we dramatically increased *Ngf* expression and also successfully depleted these cholinergic innervations. Although the cholinergic fiber density in the AAV-*Ngf* + anti-p75^NTR^Saporin group were a little bit higher than the AAV-eGFP + anti-p75^NTR^Saporin group, both groups reached 90% of cholinergic fiber depletion in the hippocampus compared to untreated WT mice. No difference in spatial memory was observed between these groups, suggesting simply increasing *Ngf* expression without cholinergic innervation of the hippocampus is not sufficient to restore spatial memory.

Here we use brain-specific *Ngf* conditional KO mice to reveal the importance of NGF in regulating hippocampal volume, behavior, synaptic plasticity, neurogenesis, and cholinergic innervation. Furthermore, we demonstrate that the effect of hippocampal NGF on spatial memory depends on an intact cholinergic system. *Ngf* gene therapy has been tested in Alzheimer’s disease patients, but no effects were found on a latest phase 2 clinical trial^47^. However, recent data from the preceding phase 1 clinical trial with identical surgical procedure, analyzing post-mortem tissue, found that the injected AAV-*Ngf* did not directly engage the target cholinergic neurons^48^. Our data provide evidence that AAV-*Ngf* may be ineffective without cholinergic reinnervation. Our study, in combination with this recent post-mortem observation, suggests that it is still too early to conclude that NGF therapy is ineffective for Alzheimer’s disease.

## Supplementary information

Supplemental material
