# Supplementary material for: The effect of nerve growth factor on supporting spatial memory depends upon hippocampal cholinergic innervation
Source: Transl Psychiatry. 2021 Mar 15;11:162. doi: 10.1038/s41398-021-01280-3 (PMC7961060; doi:10.1038/s41398-021-01280-3)
Supplement: Supplementary file 1 — Supplemental material [file 41398_2021_1280_MOESM1_ESM.pdf]

The effect of nerve growth factor on supporting spatial memory depends upon hippocampal cholinergic innervation

Supplemental information

Supplemental Figure S1.

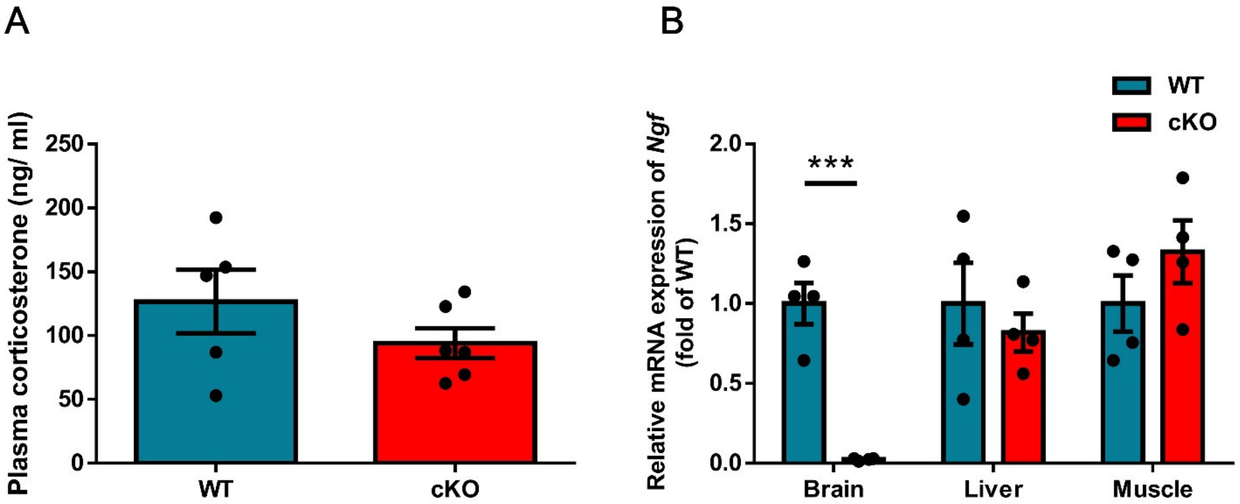

a. *Ngf* cKO mice show no difference in basal plasma corticosterone levels compared to WT mice (n = 5-6). **b.** *Ngf* mRNA levels in whole brain, liver, and muscle measured by real-time PCR (n = 4). *Ngf* is specifically deleted in brain, but not in peripheral tissue.

Supplemental Figure S2.

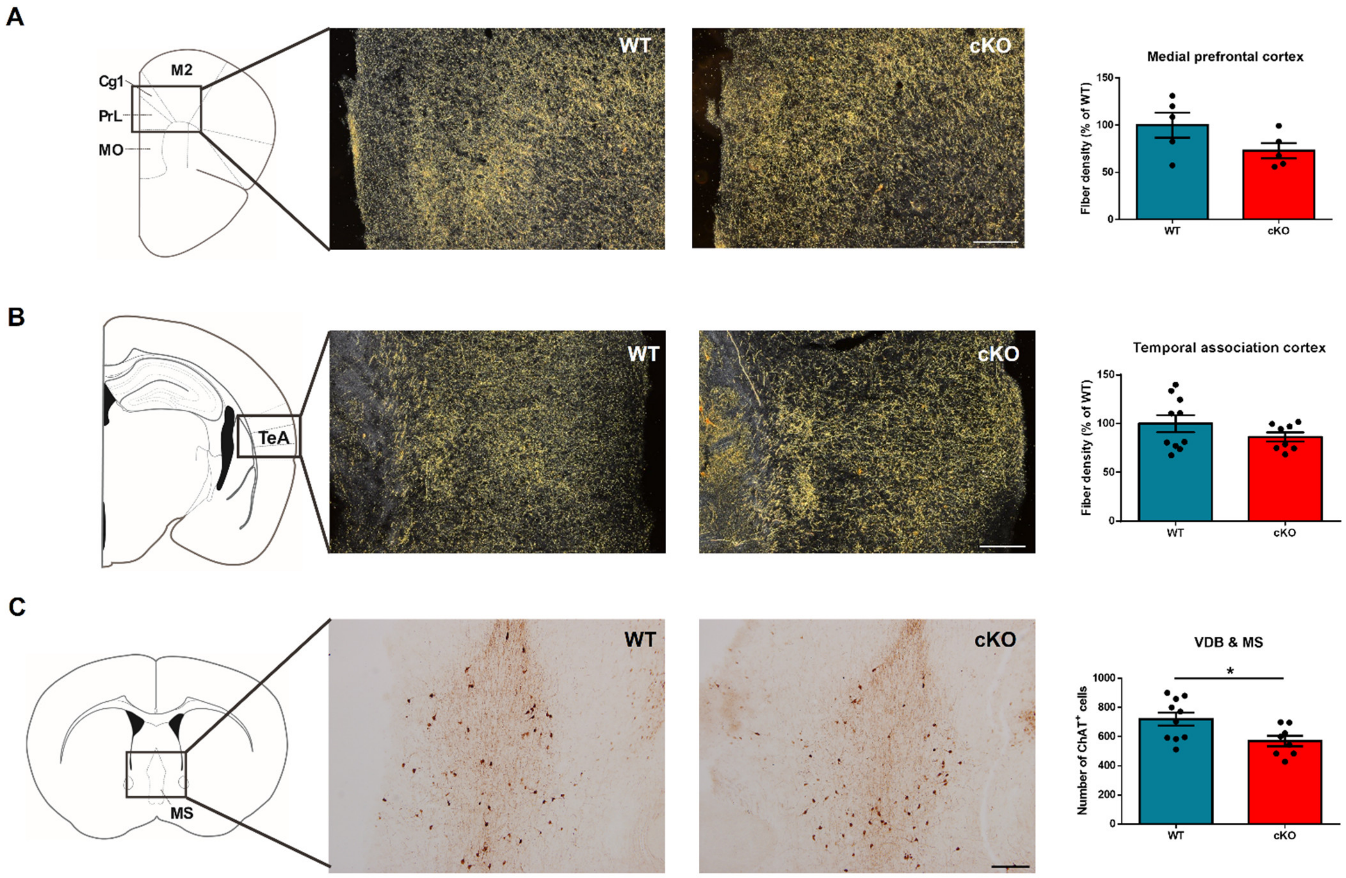

Images of ChAT staining in WT and *Ngf* cKO mice showed no difference in cholinergic fiber density in **a.** medial prefrontal cortex and **b.** temporal association cortex. **c.** *Ngf* cKO exhibited a small decrease of cholinergic neuronal cell counts in the vertical limb of the diagonal band of Broca (VDB) and medial septum (MS).
